# Scaling-Up Performance-Based Financing in Burkina Faso: From PBF to User Fees Exemption Strategic Purchasing

**DOI:** 10.34172/ijhpm.2020.209

**Published:** 2020-11-09

**Authors:** Mathieu Seppey, Valéry Ridde, Paul-André Somé

**Affiliations:** ^1^Université de Montréal, École de santé publique (ESPUM), Montréal, QC, Canada.; ^2^Research Institute for Sustainable Development, CEPED (IRDUniversité de Paris), Paris, France.; ^3^AGIR (Action-Gouvernance-IntégrationRenforcement; Groupe de travail en Santé et Développement), Ouagadougou, Burkina Faso.

**Keywords:** Scale-Up, Health Financing, Performance-Based Financing, Burkina Faso, Project Implementation

## Abstract

**Background:** Numerous countries have undertaken performance-based financing (PBF) reforms to improve quality and quantity of healthcare services. However, only few reforms have successfully managed to achieve the different scale-up phases. In Burkina Faso, a pilot project was implemented, but was put on hold before being scaled. During the writing of this article, discussions to scale-up were still ongoing on a national strategic purchasing strategy within a government led user fee exemption policy.

**Methods:** This study’s objective is to identify facilitators and barriers to scaling-up for that pilot, based on the World Health Organization’s (WHO’s) theoretical framework. Interviews were conducted in three health centres and in Ouagadougou to discuss the scale-up with different actors. The software QDA Miner© was used to help in the framework analysis.

**Results:** The low involvement of some key stakeholders (mainly decision-makers) and the unstable context hindered ownership of the project, thus its priority on the political agenda. PBF reform therefore lost its momentum to the benefit of a user fee exemption policy. This latter program was seen to be more beneficial since it addressed access to healthcare services, in comparison to service quality, which was the PBF’s relative advantage. A scale-up of some PBF elements (eg, strategic purchasing tools) is however still in discussion in 2019, but would be integrated within the user fee exemption program. Increased costs during the PBF’s implementation gave the impression that the project was too costly and not scalable. The involvement of an important funding agency (World Bank, WB) also fed the impression of high costs, which demotivated the actors, especially decision-makers.

**Conclusion:** Contextual factors remain central to the implementation of PBF, while their evaluation and mitigation have remained unclear. The participation of key actors in scaling-up operations and the use of social science as tools to better understand the context is therefore primordial.

## Background

**Key Messages**

** Implications for policy makers**

- Involvement of key stakeholders should be from the start of a project’s implementation, to favour their appropriation and prioritisation of the scale-up operations.
- Context evaluations should be performed during a project’s implementation to provide adaptation or mitigation mechanisms, due to the dynamism of the contexts.
- Social sciences theories and practices should be employed to better assess the different aspects of scale-up processes in relation to healthcare policy or projects.

** Implications for the public**

Whilst performance-based financing (PBF) was a priority for many actors in the beginning, the evolution of the burkinabè context led to changes in priorities and thus an unforeseeable future for PBF. The PBF reform was perceived as too costly and as a foreign agenda. To avoid this, more attention needs to be put on determinants of sustainability and scaling-up. National values, needs or structures must be accounted for, especially when programs are designed from abroad. While international programs still take up an important percentage of development work, the agenda must be set by national entities with international agencies playing a supportive role.

There are as many ways to finance healthcare systems as there are healthcare systems. However, a new way of financing has become ever more prominent, results-based financing (RBF). This article will provide an overview of RBF reforms as well as a theoretical background for scaling-up operations. It will then present the case of RBF in Burkina Faso, where a case study was conducted based on the ExpandNet framework for scaling-up.

###  Performance-Based Financing Reforms

 There is a wide variety of RBF programs: performance-based financing (PBF), pay-for-performance, conditional payments, etc.^1-8^ These different types of RBF programs can however be categorised according to their main focus, whether they target the demand-side of health services (eg, vouchers, conditional cash transfers) or the supply-side (eg, performance indicators, quality/quantity targets that healthcare providers have to reach); these goals are not exclusive and can be implemented conjointly.^9^ Different tool kits have been developed for RBF implementation, the main ones being developed by the World Bank (WB)^1^ and the Dutch development agency/Royal tropical institute.^10^

 More specific to African contexts, PBF represents a form of “fees for services” paid to health workers and/or organisations when targeted services are provided quantitatively or qualitatively.^11,12^ Different versions of PBF exist, however the main goal of these PBF reforms remains similar, that is to improve the quality and quantity of healthcare system services. This is done by activating different levers: greater autonomy of healthcare centres, clarified/better defined roles and responsibilities for healthcare agents, strengthened planning processes, increased community participation in the healthcare centres’ management, reinforced monitoring and evaluation system (whether independent or not) or enhanced transparency mechanisms.^1,10,13^ A major change brought by PBF reforms is however the shift from input to output payments, which makes the payments retroactive rather than prospective (eg, salaries, historical budgets).^8,14^

 PBF principles also call for a greater separation of 5 different functions in the healthcare system: providing services (eg, healthcare providers), regulating services (eg, national government), purchasing services, holding fund, and community participation (eg, management committee).^1,13^ Different PBF programs can choose to separate, or not, those functions (eg, in Mali, the Dutch development agency was the fund holder and the service purchaser, or in Burkina Faso, the government was regulator and one of its departments was the service purchaser).^15^

 PBF programs are in the spirit of the times and are used worldwide, maybe more specifically in low- and middle-income countries, such as in sub-Saharan Africa.^3,11,12,16-18^ In 2006, only 2 programs were implemented, one on a national scale (Rwanda)^19^ and another as a pilot (Burundi).^20^ Seven years later, there are 21 programs implemented (18 pilots and 3 on a national scale), while 9 other programs were in discussion in other countries.^1^ Of these pilots and programs, too few PBF projects have been scaled-up to the national policy level; such as in Rwanda, Armenia, Burundi, Cambodia, and Cameroon.^20,21^ Key enablers were found in regards to the adoption (going from a pilot to a national program) (eg, favourable policies and institutions, technical experts at the national levels, policy entrepreneurs) and the institutionalisation (going from a program to a policy) of PBF projects (eg, structures facilitating autonomy, leadership from the health sector and beyond, national financial investments).^20^

 Through the study of those cases, barriers and facilitators have been identified related to the scaling-up process, such as financial, legislative or political aspects.^20^ However, while context is central to scaling-up operations, few studies provide the in-depth contextual insights necessary to understand how the interventions reach (or not) the national policy level or even the system’s level.^22,23^ Our study in Burkina Faso will therefore aim to provide those insights and shed greater light on those mechanisms (that led to the non-accession to the national policy level).

###  Scale-up Components and Determinants

 Scaling-up has become an important part of implementation science, with the rise of evidence-based interventions (EBI) and the need to generate the best results possible from interventions.^24^ Scaling-up can be described as “the deliberate effort to broaden the delivery of an EBI with the intention of reaching larger numbers of a target audience. Often an EBI scale-up will target health delivery units within the same, or very similar settings, under which the EBI has already been tested.”^24^ New concepts can also be found such as scale-out, where scaling-up is then applied to new population and/or delivery systems.^24^

 Many conceptual frameworks could also be used to assess scaling up processes: the “*Consolidated Framework for Implementation Research,*” the theory for the diffusion of innovation or the “*Going to Full Scale*” developed by the Institute for Healthcare Improvement.^25-30^ This latter model is a consolidation of the different theories in the area of implementation and scaling-up.^30^ This framework is divided in 3 elements: scaling-up phases (preparation, development, testing, final scale-up), adoption mechanism (communication, networking, leadership, culture of urgency and persistence), and scale-up support systems (learning and information systems, infrastructure and human capability facilitating scale-up, and a sustainable design).^30^ Even more specific to PBF programs, a special journal issue was published (sponsored by the Alliance for Health Policy and Systems Research; AHPSR) to present another conceptual framework, based on a multi-country research program looking at 10 PBF programs undergoing a scaling-up process.^20,21^ In this framework, 4 phases are identified, based on case study reports for different countries: the generation of the project (from an idea), its adoption (in the form of a program), institutionalisation (into a policy) and expansion (to the system).^21^ The framework used in our study is the one developed by ExpandNet and the World Health Organization (WHO) (see below). Few reasons explain the use of this framework: its extensive use in different contexts (especially in low- and middle-income countries), its generally accepted clear definitions in regards to scaling up attributes and processes,^25,28,31,32^ its development through a network of various institutions (international organisations, non-governmental organisations [NGOs], research institutions or health ministries and organisations)^28,33^ and its larger extension to other domains in health, education or development.^34^ In addition to the use of the ExpandNet framework for the presentation of the results, the AHPSR’s framework^20,21^ will also be used in the discussion to explain further these results. The latter framework was not used for data collection or analysis since it was not published at the moment of data collection and analysis.

 Having all these different models, more case studies on scaling-up PBF programs could help improve conceptual comprehension of the phenomena and further develop the abovementioned analytical frameworks.^21,26^ The assessment of PBF scale-ups is also very interesting due to the number of PBF reforms around the world, mostly in low- and middle-income countries.^3,11,12,16-18^ Furthermore, without many examples of a PBF institutionalisation phase, the case of Burkina Faso could provide many empirical insights that would be pertinent to both the understanding of the scale-up concept and providing concrete evidence in the case of PBF.^20,26^

###  Performance-Based Financing in Burkina Faso

 Burkina Faso has a certain experience in attempts to contractual approaches in its health system such as performance contracts in health districts or the contractual process with NGOs and other associations.^35^ Its following its experiments that the WB proposed to the Ministry of Health (MoH) to test a first important PBF program (totally funded by the WB) in 2011 with 3 health districts.^35^ That program was a pilot project, whilst the subsequent project, which is the object of our research, was not yet planned. Its name changed from pilot to pre-pilot once the latter could be put in place. The pre-pilot did not benefit from any independent evaluation, neither from an impact evaluation, but enabled to test different tools and to emulate the PBF idea. An evaluation from a national consultant paid by the WB was conducted, notably on implementing processes.^35^ This evaluation on care utilisation was not rigorous enough to provide with tangible results, due to the small scale of the evaluation and its study design.^36^ The WB therefore gave to another consultant the mandate to assess the pre-pilot’s effects, more precisely on care utilisation, with a time series design.^37^ The state and the WB’s decision to extend PBF to an additional 12 districts in 6 different regions in 2013 was taken before the publication of this evaluation.^38,39^ In total, the new pilot would concern 561 primary healthcare centres (CSPS:* Centre de Santé et de Promotion Sociale*), 15 second line healthcare centres (11 district: CMA [*Centre Médical avec Antenne chirurgicale*] and 4 regional: CHR [*Centre Hôspitalier Régional*] hospitals).^39,40^ Its main objective was to strengthen the national healthcare system’s performance by improving the quantity and quality of healthcare services, by supporting the monitoring and evaluation system and by consolidating partnerships with the different communities, healthcare agents and private organisations.^40^ Four types of PBF were tested in Burkina Faso^36^:

- PBF1 consisted in conventional PBF where health centres were financed by the WB, through the *Programme d’Appui au Développement Sanitaire* (PADS), according to their healthcare services’ quantity and quality. The quantity and quality of services were assessed every 3 months by external verification and control agencies (ACV: *Agences de contractualisation et de vérification des services*). User fees remained for incoming patients.
- PBF2 consisted in PBF1 with the addition of a subsidisation for the poor’s healthcare services. User fee exemptions were given to the poorest 15%-20% of the population, which were selected through a community-based process. Healthcare services provided for this population were paid at higher price to palliate the user fee exemption.
- PBF3 consisted in PBF2 with the addition of a financial motivation for the healthcare providers, when working with the poor (the selected 15%-20%). Services provided to the poor are paid more than in PBF3.
- PBF4 consisted in PBF1 with a community-based health insurance. Insurance premiums for the poor (the selected 15%-20%) were subsidised, and services provided to them were paid at higher price such as in PBF3. Capitation payments replaced user fees for the general population.

Those 4 types of PBF implied many similar aspects such as the service targeting by the MoH (playing the role of regulator) and its national, regional and district directions. The different health centres (playing the role of service providers) then supplied services in relation to the set targets and needs of the community. Qualitative and quantitative evaluations are made by ACV which will judge if services can be purchased or not by the PADS.^38-40^

 The reasons for this extension remain unknown and are not the object of this article. It could be possible the WB wished to pursue its tests in the region even without available data. Being limited in its resources, the country might have accepted that proposition due to the importance of the project’s budget and inputs it could bring to the health system and to the health professionals. The question of ownership of such health policy in Africa, and of PBF especially, is at the centre of actual debates.^41,42^ Like in Mali,^15,41^ multiple strategies were implemented by the WB to influence the decisional process and to reach acceptance of the project: study tours, consultations, supplementary budgets, workshops, etc.

###  User Fee Exemption Policy

 The user fee exemption policy started in April 2016 with the objective to increase the financial access to certain populations (eg, children under 5, pregnant/post-partum women) or services (eg, family planning, emergency obstetrical care) for an estimated 5 millions beneficiaries.^43-45^Many actors were involved in the design such as healthcare centres (from all levels), different directions from the MoH, NGOs (such as international [HELP, Terre des Hommes, Action Contre la Faim] or national organisations [Association Songui Manegré/Aide au Développement Endogène, ASMADE]) and the Ministry of economy and finance (MoEF).^44^ The idea of a user fee exemption program emerged in 2006 with a first pilot implemented in 2008 by a consortium of the different NGOs above-mentioned. The program was then implemented nationally in 2016. The *Caisse nationale d’assurance maladie universelle* was established in April 2018 and is now being transferred the responsibilities of the program from the MoH.^45^

 The objective of this research is therefore to better understand the processes of the scaling-up of the PBF pilot (not the pre-pilot) in Burkina Faso. This article will answer the questions of what are the facilitators and barriers to scaling-up for that pilot, and why the program was not scaled-up in the end.

## Methods

 This study’s design is a case study nested in a larger longitudinal and multiple case study.^46^ This methodology was chosen to study the scaling-up potential of the intervention because this concept can be highly complex and be perceived differently from the perspectives of different stakeholders.^47^ The choice of using a case study design is pertinent since the assessed intervention is a phenomenon that is anchored in a real and specific context (a program implemented in Burkina Faso by different actors)^47^ and that a holistic and comprehensive picture (via triangulation of sources and data collection methods) of the program is needed, through the analysis of the lived experience, challenges or impacts of/on the different stakeholders.^47,48^

###  Conceptual Framework

 The conceptual framework used for the study is the one presented by the WHO and the ExpandNet network, which was developed earlier than those presented above.^28^ This framework divides the concept of scaling-up as 5 main elements: (1) the innovation (the PBF pilot project), (2) the host organisation adopting the innovation (Burkina Faso’s healthcare system), (3) the resource team (the PBF cell, however, not being clearly identified as such), (4) the scaling-up strategies (different attributes possible), and (5) the general context of implementation (socio-cultural, politico-economic, etc). For each of these 5 elements, different attributes were assessed to provide a comprehensive picture of the scale-up situation in regards to the PBF pilot program in Burkina Faso. The most important attributes are therefore presented in the result section and are further described in the work of Simmons et al.^28^

###  Data Collection

####  Sites

 This scale-up assessment took place within a larger, longitudinal and multiple case study in 3 healthcare districts (Diébougou, Ouahigouya, and Solenzo).^36^ Those districts were selected based on the criteria of representativeness of the different contexts within the country (districts represented different regions: respectively Southwest, Northern and Boucle du Mouhoun regions). From these districts, 6 CSPS and one CMA or CHR served as cases. These healthcare centres were selected according to the type of PBF being implemented and their level of performance linked to service provision before the intervention (good, fair, poor).^36,49^ Performance was assessed in relation to service provision indicators (eg, vaccination coverage, number of consultations). For this study on scaling-up, 3 sites were used from the larger sampling of 21 sites (this is due to time/resource constraints). Those sites were selected based on the criteria of security, physical access, and presence of knowledgeable participants. The selection was made with the local research team members in charge of the different districts. Also, since any scale-up operation requires a major involvement of stakeholders at the national level, members of different organisations were consulted in Ouagadougou.

####  Participants

 Within the 3 healthcare centres (2 CSPS and one CMA), from 4 to 8 participants were interviewed by MS. They consisted of a variety of actors: healthcare practitioners from different levels (community healthcare worker[*agent itinérant de santé*], midwives, nurses, head nurses [*infirmier chef de poste*: ICP] or head doctors[*médecin chef de district*: MCD]), senior-managers, management committees (*comité de gestion*: COGES) (eg, presidents, treasurers), and representatives of the local health insurance program (ASMADE) (eg, social coordinators, people in charge of the implementation or communication).

 At the national level, MS further extended the data collection phase to interview different members of 4 other functional branches of the PBF project: (1) service purchasers (the PADS) (which aim to help the implementation of interventions at the operational/national level), (2) implementers (PBF coordinating cell: *cellule FBR*), (3) ACV, and (4) regulators/decision-makers from different divisions within Burkina Faso’s MoH. External experts (NGOs, WB) were also contacted to provide another perspective on the issue (Table 1). These participants were purposely selected according to their level of knowledge or involvement in the PBF project. They therefore represented an exhaustive picture of the different stakeholders taking part in the intervention and helped reach data saturation point (N = 37).

**Table 1:** Participants Sampling

**Local Level **	Healthcare agents	8
	Head-managers (ICP + MCD)	3
	COGES	3
	Health insurance (ASMADE)	4^a^
	Unions	1^b^
**National Level**	Service purchaser (PADS)	3
	Implementers (PBF cell)	4
	Senior-managers (*Directions générales de la santé, de la protection sociale-promotion et mutuelle, des études et des statistiques sectorielles et des établissements de la santé*)	6^c^
	ACV	2^d^
	External experts	3
**Total of Interviews**		37

Abbreviations: ICP, *infirmier chef de poste; *MCD, *médecin chef de district; * COGES, *comité de gestion*; ASMADE, Association Songui Manegré/Aide au Développement Endogène; PADS, *Programme d’Appui au Développement Sanitaire; *PBF, performance-based financing; ACV, *Agences de contractualisation et de vérification des services.* ^a^ One participant was previously also part of the COGES. ^b^ One participant was also a member of the healthcare agents. ^c^ Three of these participants also had positions in other divisions linked to this PBF implementation. ^d^ One interview was with both the director and an agent of an ACV.

####  Data and Analysis

 Data collection took place in 2 phases, the first being at the local level (22/10/2016 to the 3/11/2016) and the second in the capital city (9/11/2016 to the 20/11/2016). Interviews were semi-structured and were conducted (all interviews were conducted by MS, apart from 2 interviews conducted by trained research assistants) with the help of an interview guide based on the ExpandNet/WHO scale-up framework.^50^ The guide’s themes covered the different elements of the framework (eg, attributes of the intervention, health system, strategies) and facilitated the subsequent analysis which was both deductive and inductive. While maintaining the themes, the guide’s questions evolved during the data collection phase to become more precise, in regards to the roles of the participants and the missing information. Interviews were mainly conducted at the participants’ workplace as well as their homes (only at the local level), depending on their preference. They were conducted in French (exception of one interview in Dioula), lasted from 30 minutes to more than 2 hours and were audio-recorded and transcribed into *verbatim*. Observation notes were taken during data collection which enabled the gathering of more informal discourses related to the scaling-up processes of the PBF project. Once transcribed by trained research assistants, *verbatim* were re-read with the audio-recordings (MS), re-transcribed if needed, put together with the software QDA Miner© and coded (MS), according to the conceptual framework. The framework analysis that was conducted (MS) can be resumed in the familiarisation of the data, the thematic analysis through coding (with both deductive themes/codes from the ExpandNet’s framework and inductive themes/codes emerging from the data), indexing codes to all data and finally charting the data according to their indexes.^50,51^

## Results

 This study’s results will be presented according to the ExpandNet framework, in regards to the attributes of its different elements: (1) the project, (2) the healthcare system, (3) the resource team, (4) the scaling-up strategies, and (5) the general context of implementation.^28^

###  PBF Project’s Attributes

####  Credibility

 The interventions’ credibility was mainly based on previous successful PBF reforms such as those in Rwanda, the Democratic Republic of Congo and Burundi, the later which was also coupled with a user fee exemption policy: “*We *[external burkinabè expert] *had experiences from other countries. Most of the time, the experiences showed that there were results which were much more positive than negative.*” (External expert #2, Ouagadoudou)^[1]^; “*We told ourselves: if it has worked elsewhere, it can also work here.*” (Statistician, healthcare centre #3). However, for some healthcare providers, PBF was somehow discredited due to certain implementation problems such as delays in payments, which resulted in a certain lack of trust: “*If we manage to pay the 6 late months, and the next months to follow the subsides, thus… the people will get better confidence*” (PBF cell member #4, Ouagadoudou).

####  Observability

 Outcomes of the PBF project were interpreted differently at the local than at the national level: “*We *[local implementers]* see the effects it *[PBF]* produces, the advantages it generates. Since we are direct actors, we implement, we have seen what it brought to us. This is thus sufficient to say that it should be a priority. However, the decision-makers don’t implement… maybe it doesn’t allow them to better appreciate it, to make them say it should be a priority*” (MCD, healthcare centre #3). In regards to decision-makers, more negative or a lack of outcomes related to implementation problems were perceived, such as payment delays, strikes or lack of investments: “*I, myself, have visited Cameroun in the PBF centres. We have seen renovated offices, beds, mattresses … When we arrive, it is visible, perceptible. Then here, even if people do it *[PBF]*, it is on paper, in regards to investments… When we arrive like that, we don’t have the impression that there were renovations*” (DGESS representative, Ouagadoudou). “*It’s already not evident with 15 districts … When I have feedbacks, complaints from district teams because payments are not regular… if you are in this dynamic, it is not evident that we want to pass to scale*” (DGS representative, Ouagadoudou).

####  Relative Advantages

 The relative advantages of the intervention were well understood in theory. PBF was thought to be effective in regards to improving quality, strengthening the monitoring and evaluation system, motivating healthcare practitioners or reporting. This relative advantage was however often related to the user fee exemption program, as a complementary reform: “*Now, we can readjust the user fee exemption and add certain elements of PBF, such as the verification*” (External expert #3, Ouagadoudou). “*User fee exemption will act on the quantity side whilst PBF will act on the quality side*” (PBF cell member #1, Ouagadoudou). On the other hand, in practice, people at the decisional level were not moved by those arguments: “*If the Ministry *[MoH]* had understood the added value of PBF, it would have been them that would have ran after us*” (PBF cell member #2, Ouagadoudou).

####  Easiness to Install/Testable

 The PBF intervention was somewhat complex to install and to comprehend due to the 4 different types of PBF implemented. Many modifications and adaptations also hindered implementation: changes in indicators, delays in payments, etc. The pilot project was nonetheless testable and even benefitted from a first scale-up operation, bringing the project from 3 health districts to 15. During its implementation, the project did however face different unintended and undesirable effects that were not always addressed.^52^

####  Compatibility

 PBF ran counter to the national healthcare system’s functioning in many aspects: the use of retroactive financing (*versus* inputs-based/prospective financing) or the autonomisation of the healthcare centres in regards to their spending. For some actors, mainly the syndicate, this reform meant privatising the burkinabè healthcare system by decentralising the spending powers and externalising some activities (eg, quality/quantity verifications). “*Do we not want to privatise our health services?... If we are in the public function, we have a salary and it is normalised. I am a nurse with three years of seniority, my salary is known. Can it be different from one centre to another? This is not possible*” (External expert #1, Ouagadoudou).

###  Burkinabè’s Health System Attributes

####  Implementation Capacity

 The implementation capacity for healthcare centres was mostly perceived as adequate since the health system in Burkina Faso was considered functional: “*When you take the examples of countries such as the Democratic Republic of Congo or Burundi where the healthcare system wasn’t necessarily performant, PBF proved itself rapidly. But here, PBF came to find out that it worked. In reality, it’s the resources that are lacking, apart from that, the healthcare system worked*” (PBF cell member #4, Ouagadoudou). PBF activities also seemed similar to daily routines already in place and appropriate for the different sizes or types of healthcare centres: “*With the activities it *[PBF]* does here… I think that it will function very well… There is no problem, since it functions according to the size of the CSPS*” (ICP, healthcare centre #1).

 However the equity between centres was an issue for certain: “*There must be a minimum and I think there are centres that don’t have this minimum. I’m talking about human resources, materials*” (External expert #2, Ouagadoudou); the incapacity of poorer healthcare centres to maximise the benefits of State resources (eg, staffing or other materials) seemed inequitable. To provide an equal minimum level of investment everywhere was perceived as a prerequisite since those investments were public funds.

 At the State capacity level, few actors seemed positive about an eventual scale-up of the intervention as the project stood due to the amount of funding needed. Changes had to be made, especially in regards to the expensive verification phase: “*We must prioritise the criteria *[for evaluation]* and then lighten the control and evaluation system. I think that then we’ll be able to manage the budget to go to scale. Actually, PBF is much money*” (External expert #2, Ouagadoudou). However, the majority of participants suggested the PBF reform was too expensive to scale-up: “*When we illustrated all the financial realities, we felt the people went cold*” (DSF representative, Ouagadoudou); “*I remember a meeting with the Ministry of economy and finance *[MoEF]* where its representative said: ‘You with your PBF story! Burkina Faso isn’t Rwanda. What do you think? Everything you’re telling me here, you think we can do that in Burkina? We can’t!’*” (External expert #1, Ouagadoudou). Partnerships with donors were evoked, showing the incapacity of the State to rely on itself: “*Scale-up by the State should depend on partners because it has no means for it*” (External expert #2, Ouagadoudou). It is worth noting that this issue was thought of during the implementation process, but was never addressed: “*We must say that during the implementation phase, it was always a preoccupation, to see how we’ll do for the scale-up. Now, where to find the money for the scale-up remains the big question that remained pending*” (DGESS representative, Ouagadoudou).

 Social acceptability and legal grounds of the scale-up were also mentioned in regards to inequitable treatment favouring the health sector: “*The common basket, it’s naturally the State budget. If we have to treat a sector differently from the others, we create useless conflicts… There is a law that is about the public function of the State. Everyone gets to be treated the same. Why doctors should be treated differently from the teachers, or the police, or the one working at the MoEF ?*” (DGS representative, Ouagadoudou).

####  Leadership and Internal Advocacy

 Stakeholders’ level of enthusiasm towards the scaling-up process was different whether stakeholders were from the local level, with a more operational stance, or at the national level with a decisional role. Implementers, healthcare providers and managers had personal or institutional interests in the reform’s scale-up: “*It *[PBF]* allows us to have bonuses outside of the revenues of the drugs’ sales… it allows us to have resources to function without too many difficulties*” (Statistician, health centre #3).

 From a decisional point of view, the PBF intervention was very politicised, which created “*dogmatic*” points of views, with PBF people being either “*pro*” or “*con.*” Arguments were therefore developed against its scale-up operation: “*There are people that are fundamentally not in tune with PBF because, according to them, PBF pays people *[eg, healthcare providers]* that were already paid by the State to do the activities. Therefore, from an ethical point of view, it is not much… It’s also the workload that needs many more forms filled out, much time to spend with the patient*” (PBF cell member #4, Ouagadoudou). In addition, the reformative aspect of the scale-up operation was perceived as an important political change for the national financing, since it would entail a certain form of decentralisation:

 “*We *[Health centres national division: DÉS] *imagined that the MoH could negotiate with the MoEF to make the financing of the healthcare sector, anyway of the healthcare centres, to be not much input based… but to purchase performance. But this negotiation did not succeed because the MoEF said: No, if we grant you this ease, other ministries will ask the same, we risk not being able to get out of it*” (DGS representative, Ouagadoudou).

 By not making PBF a government priority, other stakeholders could also be influenced and reduce their engagement in the project, such as the WB that financed the pilot: “*If there are no real political will to invest, engage *[in PBF]*, I think the WB can have difficulties engaging itself*” (DGESS representative, Ouagadoudou). Furthermore, better government engagement for the PBF scale-up did not seem plausible in any foreseen future, therefore closing the window of opportunity: “*We delay the deadlines, while we try to analyse the evolution of the State’s budget. I don’t see better perspectives for the next 2-3 years to come. Hence now, what do we do? We say no… we try to progress, caracoling, from left to right, to then find ourselves in front of reality*” (DSF representative, Ouagadoudou).

###  Attributes of the Scale-up Team

 At the end of our data collection, no team was identified to conduct the scale-up operation: “*We [DGESS, Direction Générale des Études et des Statistiques Sectorielles ] still do not have a team in place for the scale-up. Like I said, it is conditioned by the partners’ availability to accompany… since at the State budget level, there is no perspective as such*” (DGESS representative, Ouagadoudou).Not being a high priority for the government, the PBF’s scale-up was on hold and reflexions were being done as how to include PBF components within the user fee exemption. The PBF cell, which was in charge of the implementation, would be the best placed team for the scale-up, but has not been clearly identified as such, the scale-up being on hold.

###  Scale-up Strategies

####  Guided Strategy

 What has been mentioned as a potential type of scaling-up strategy was mostly guided by the government, the national healthcare system and the implementers from the PBF cell. While the expansion of PBF, as it is, to other districts seems improbable as illustrated above, PBF could be institutionalised (vertical scale-up) through a national strategy, with the user fee exemption program: “*The national financing strategy for health is thinking about it now. I’m taking part in this committee. It is the financing system that will change while keeping the PBF dispositive*” (PBF cell member #1, Ouagadoudou).

####  Dissemination

 Various strategies of dissemination were used to favour the implementation of the PBF project, such as workshops, meetings, presentations of tool kits and other documents. There were fewer strategies concerning an eventual scale-up and it seemed more indefinite: “*We planned to meet up to exchange… we might go to Burundi. It seems to me that they were able to align PBF and user fee exemptions. We’re going to look and see how they did and it will consolidate our decision-making*” (DÉS representative, Ouagadoudou). Nonetheless, the dissemination strategies did not reach a significant number of key stakeholders and that there was a lack of entrepreneurship towards the promotion of PBF.

####  Organisational Choices

 For an eventual scale-up strategy, new partnerships were to be assessed: “*We want to meet all the partners, UNICEF [the United Nations Children’s Fund], the AFD [ Agence Française de Développement ], the WHO, UNFPA [United Nations Population Fund], the WB to see how we will do… to find money to finance PBF, to continue and maybe go to scale*” (PADS member #1, Ouagadoudou). However, not being a priority for the government, which prioritised the user fee exemption, the engagement of other partners towards PBF seemed lower: “*The question of appropriation *[of PBF by the government] *isn’t still solved. It is because of that I say we can’t finance PBF anymore in an independent manner like we did. The environment we are in now, with the decision to go forward with the user fee exemption, makes that we should necessarily accompany the government with the user fee exemption*” (External expert #3, Ouagadoudou).

 Some adaptive strategies were discussed in interviews to hypothetically go forward with a scale-up. For example, the evaluation tools could be changed by adding/removing some indicators or by raising/lowering prices to obtain a more appropriate amount of money to be paid to the healthcare centres (according to the allocated budget). These adaptive strategies were employed for the first scale-up in 2013, and can still be used to reduce expenses: “*Actually, to do the quantity evaluation, we plan 1 healthcare centre /day, it is according to the number of indicators. If the number of indicators would be less, we could program two healthcare centres /day and it would reduce the cost*” (PBF cell member #4, Ouagadoudou). However, for PBF to function, core elements cannot be changed and can create important financial or technical issues: “*To involve all districts will be heavy on the central level … to be able to do the supervision … especially at the verification level, if we have a lot of CSPS to involve… because we have about 1700 CSPS … I admit it won’t really be easy to maintain a quality work*” (DGESS representative, Ouagadoudou).

 Some actors considered the magnitude of a national scale-up and they mentioned the necessary steps to take towards an incremental scaling-up: “*It will be difficult to involve every healthcare district at the same time. Maybe we could plan to expand to 50% and then go beyond*” (DGESS representative, Ouagadoudou). However, this gradual approach was in contradiction with the MoH principle, which was accountable to the whole nation in regards to the provision of equal healthcare services and their improved quality.

 Finally, a more donor (WB) driven strategy seemed to emerge from the discussions. This could be in reaction to the lower involvement of the MoH and the MoEF, the later not being clearly in favour of PBF’s principles, such as decentralising spending powers: “*The MoH is blocked by the MoEF, who says that the State’s funding is there. That’s the budget*” (External expert #3, Ouagadoudou). The PBF cell was at the centre of the project’s implementation and was thought to be the appropriate vehicle to implement the scaling-up: “*The team is already in place, it’s the team of the cell, even the PADS team*” (External expert #3, Ouagadoudou). However, this team remained within a department of the Direction Générale de la Santé (DGS), a section outside the decision-making sphere of the MoH. No information was given in regards to a participatory approach were non-experts would have a say in the scaling-up process.

####  Costs/Resource Mobilisation

 The assessment of the cost of the scale-up operation was not mentioned clearly, but an overall impression of the costs was expressed by some actors: “*It can cost a lot… it will cost a lot!*” (DGS representative, Ouagadoudou); “*It’s about one million dollars you see, per trimester!*” (PADS member #2, Ouagadoudou). Economy of scale was not mentioned, but mobilising funds was thought possible through partnerships with donors or another program, such as the user fee exemption. To our knowledge, there was neither a cost-effectiveness study nor a study of the cost for the expansion and no other funding agency has been identified or at the discussion table concerning PBF.

####  Monitoring and Evaluation

 Monitoring data was collected in relation to the payments, but because of the PBF and the integration of healthcare centres budgets, difficulties arose in regards to the analysis of the different investments made by those payments; therefore the effects of the project. International partners (Centre Muraz and the University of Heidelberg) were involved at the end of the project’s impact evaluation. Since PBF was not a high priority for the government, the probability of scaling-up was highly dependent on this evaluation: “*The Centre Muraz is doing the impact evaluation now. We’ll see if it works or if we go for a failure*” (External expert #3, Ouagadoudou). While the impact evaluation seemed very important to sustain/scale-up PBF, punctual problems during the implementation phase already negatively affected the opinion of some decision-makers, therefore reducing the prospect of PBF’s scaling-up. In addition, while the impact evaluation was carried out at the end of 2017, these results are still not available nor shared in May 2019.

###  Context

 Political instability also did not allow a favourable context for PBF’s scale-up: “*Today, I *[PBF cell]* can say that it *[PBF]* is always a priority, but we understand that with the upheaval that we had, the insurrection *[2014]*, there is a form of instability at the political level … so that it’s a bit slower *[PBF prioritisation]” (PBF cell member #3, Ouagadoudou). It is important to note that the reform was more highly prioritised at its beginning: “*In 2010, when we *[PBF Cell]* installed ourselves, it was perceived as a priority … because the Minister came to participate in our workshops*” (PBF cell member #3, Ouagadoudou). However, this prioritisation changed due to much instability in the government: “*From 2011 to 2016 there were at least five Ministers, thus it wasn’t a kind of help for PBF, because people change and priorities are not the same. For example, those that were at the start of PBF, if they were always there, PBF would have even more flown away*” (PBF cell member #4, Ouagadoudou). The presence of the project in the governmental agenda therefore seemed to be dependent on specific high-level individuals advocating for it, which made the support for the scale-up much weaker.

## Discussion

 While results show that the potential may be weak for an eventual scaling-up of the PBF pilot, it is hard to speak of a future scale-up for PBF. A potential hypothesis would be to point out the lack of ownership of the program by actors at the government level, more especially within the MoH.^41^ Firstly, the government decided not to allocate any financial resource to the program, which was then tested on the sole subvention of the WB. Furthermore, the program was not given priority politically, to a point where the PBF’s implementation cell became isolated within a smaller department of the DGS without managing or authority levers. Members of the cell often complained about that situation and many of them did not remain to pursue a career at the regional or international level promoting PBF. At the moment, PBF has ceased since 3 years and no sign is showing that the state will pursue the program. Independent evaluations showed challenges in the implementation,^53^ issues with unexpected effects^52^ or the lack of effectiveness in regards to maternal and child health indicators.^54^ An impact evaluation paid by the WB has been conducted with mitigated results, this explaining maybe why it was not officially shared to the MoH or not publicly released after 2 years from data collection. No study of the costs seems to have been done. The WB is now trying to bring strategic purchasing to the discussion table with the state.

 Talks are underway aiming at another axis of intervention with the objective of insuring financial accessibility to healthcare, which is also in the *Plan National de Développement Sanitaire 2011-2020 *(PNDS).^55^ Both the supply-side (eg, PBF) and the demand-side (eg, user fees exemption) of healthcare were accounted for in the PNDS, but access to services (demand-side) seems now to prevail. Concerning the *Stratégie Nationale de Financement de la Santé pour la couverture sanitaire universelle 2018-2030* (SNFS), very few references are made to PBF, which seems to have been replaced by strategic purchasing of services, the third axis of the financial plan.^56^ Strategic purchasing will be possible through a framework for monitoring performance, which will link allocated resources to program results. Incentives for performance are also evocated very briefly without reference to PBF. It is interesting to note that PBF’s mechanisms and goals are still present, in some ways, in this SNFS.^56^

 According to a more recent (published after our data collection) and specific framework (based on PBF interventions),^21^ the Burkina Faso case would aim simultaneously to adopt and institutionalise PBF (phase 2 and 3, respectively). Adoption would refer to the elaboration of national operational tools (eg, guidelines, contracts, job descriptions) and adaptive institutional measures leading to a greater population and/or service coverage.^21^ Institutionalisation of the PBF program could be through its integration into a national policy (eg, User fee exemption or universal health insurance), leading to the MoH’s objectives.^21,55,57^ Being concurrently in both PBF’s adoption and institutionalisation could be caused by the prioritisation of another national program (user fee exemption) and therefore, the need for PBF’s advocates to get on the fast track and join the user fee exemption at the policy levels.

###  Adoption

 Many enabling factors and barriers have been identified in regards to those 2 phases and can provide a comparison basis between Burkina Faso and other countries (Table 2). Concerning the burkinabè experience, few enablers were present, apart from actors with technical capacity. Pre-existing institutions and policies were not naturally designed to implement a PBF reform, mainly due to the centralisation of the healthcare system. In comparison to the burkinabè experience, the Malian healthcare system provided a more enabling environment by being highly decentralised and consequently by giving its *associations de santé communautaires* (Community healthcare association, similar to COGES) the decisional power to invest in healthcare centres; investments were perceived very positively and cash transfers were not difficult, apart from delays.^15^ In Burkina Faso, the centralised healthcare system did not provide such an environment, which later served as an argument against PBF, namely, that local improvements were not observable because of the rigidity in fund transfers and low investments in resources. PBF also brought to light the issue of transparency in a context of social instability (2014 insurrection and demand for better governance). Delays and incomprehension regarding the payments were perceived as a lack of transparency and could have negatively depicted the project; the contrary of an enabling factor.^20^ Like the Chadian experience where PBF’s adoption failed, Burkina Faso’s pilot lacked committed policy entrepreneurs.^58^ Being the principal advocate for PBF, the implementation cell would have benefited if it were more directly linked to decision-making actors, such as the MoH or the general secretary. A similar situation happened in Chad, where the PBF pilot was within the Ministry of Economics and International Cooperation and not directly linked to the MoH. As a result, ownership or buy-in by national authorities (MoH) remained low for both countries.^20,58^ This can be explained by the important amount of funding that needed to be mobilised and the importance for donors to better monitor activities, which is critical in any PBF design.^41^ The MoH’s engagement in the pilot could have been greater from the start, which could have facilitated the transition from a pilot project to a national program (managed by the state rather than the WB for the pilot), like in the Cameroonian experience.^59^ Public providers also could have been more engaged in the pilot, to ensure a better comprehension of its mechanisms and to reduce the perception of a privatisation of the healthcare system. Finally, the “scale-out”^24^ or “roll-out”^21^ of PBF (specifically to scale PBF to the whole population) was perceived as being too costly for the national government to implement. Such reactions to PBF were also seen in Malawi, where only personnel costs accounted for 47% of the total costs for the intervention, which was higher than the costs of incentives (34%).^60^ It is worth noting that in the Malawi case, the former cost was mainly during the design phase (80% of the total cost of the intervention), but still was 30% during the implementation. The total amount for the design phase also corresponds to more or less a third of the intervention’s costs.^60^ This situation, in addition to poor planning in the case of Burkina Faso, might have negatively influenced perceptions about the whole project, hence leading to a change of priorities towards strategic purchasing.

**Table 2:** Burkina Faso’s Case in Relation to Shroff et al^20^

**Factors**	**Phases**	
	**Adoption**	**Institutionalisation**
Facilitators	- Pre-existing favourable institutions and policies - Pro-transparency context - Pool of actors with PBF technical capacities - Dedicated policy entrepreneurs	- Structures facilitating autonomy - Technical leadership from the PBF cell and external actors
Barriers	- Pilot’s implementation without enough MoH involvement leading to low appropriation - Local organisations (public sector) felt left out at the design and implementation phases leading to misinterpretations - Impression of too high of a cost to scale-up - No evidence about its effectiveness	- Low political engagement with key stakeholders - Absence of national resources involved - Low ownership of the program

Abbreviations:PBF, performance-based financing; MoH, Ministry of Health.

###  Institutionalisation

 In regards to the institutionalisation phase and national financing, discussion of a PBF scaling-up seemed dependant on external donor-partnerships. In Benin, PBF was scaled-up nationwide and was funded through Gavi, the Global Fund to Fight Aids, Tuberculosis and Malaria or Enabel (Belgian development agency), which adopted a similar approach to PBF as in Burkina Faso, with the WB.^61,62^ After the scale-up, PBF’s sustainability in Benin was at risk (and stopped) due to different factors, 3 being the lack of effectiveness for healthcare utilization, the lack of integration within the national healthcare system and insufficient resources from the government (after donors pulled out). This is therefore contrasting with other experiences in Cambodia or Armenia, where national funding was promoted, thus enabling ownership of the scale-up.^20,64,64^ By not dedicating PBF a priority in the national budget, partners can be reluctant to engage in the scale-up; the strategic orientation linked to PBF (#8: the increasing of healthcare financing) only accounts for 7.6% of the entire PNDS.^55^ However, an eventuality for Burkina Faso could be to follow the steps of Burundi, that allocated public funding for PBF, but within a policy of user fee exemption for children under 5 years old and pregnant women.^20^ This program being the priority of the government, ownership could be more easily obtained, by association. Insufficient political engagement was also noted in Burkina Faso, where the PBF mainly located at a low decisional level (but still within the MoH). The weak link of the PBF cell to the MoH or the MoEF rendered communication difficult and did not enable those 2 stakeholders to appropriate for themselves the reform in the making. Conclusions from another study in Burkina Faso refer to similar barriers to the institutionalisation of a national healthcare information system, such as the lack of coordination between the various actors or a lack of participation in regards to reporting/results dissemination activities.^65^ A similar situation appeared in Tanzania, where the PBF program was “confined to a small group” within the MoH, which created a barrier in the future scale-up^20^ (Table 2).

###  Lessons Learnt 

 A first lesson from this study would be to ensure the participation of key actors (eg, local/national authorities) during the different stages of the project’s implementation; this aligns with the findings of other studies such as in Mali, Tanzania, Chad, and Mozambique.^15,20,58,66^ Projects can easily be implemented through a project-based approach, including fewer actors who can more efficiently make decisions and ensure the project’s good performance. However, in regards to scaling-up, or sustainability more generally, a new way of implementing projects should be put forward by further interacting with local/national actors, building trust and creating long term and more authentic partnerships.^67-69^ Ensuring a more participative and home-grown approach to scaling-up (or project implementation) could then reduce actual flaws in the project implementation field, such as national policy/priority distortions or undue pressure towards aspects of a project’s implementation.^70-72^

 A second lesson would be to give a greater account to the implementation’s context within which the project is taking place.^23,73^ Different frameworks have been developed in relation to the relationship between project implementation and context.^15,27,74,75^ Like the situation in Burkina Faso, the context’s influence on a project’s implementation is too important to be ignored, even more when it is a question of national policies or scaled-up projects. Political, social, cultural or economic aspects should therefore be assessed and accounted for to better understand their potential impacts on projects, programs and policies.^75^ The greater contribution of social sciences (through theories and practices) in implementation research is hence the key to improving our comprehension of healthcare policies and projects.^76,77^

 As mentioned above, many similarities were found between the scale-up experience in Burkina Faso and those in other countries (maybe more with other sub-Saharan African countries, such as Mali or Chad). Passing from PBF’s theory of change to practice is a challenge which is highly affected by the implementation context.^73^ Many examples can be used to illustrate this phenomenon in the Burkina Faso case. In theory, PBF could be applied in a wide range of situations, but preconditions still remain for a more optimal implementation and scale-up: a minimum level of decentralisation, accurate data representing the healthcare system or a perspective of implementation integrating PBF in this system.^73^ In Burkina Faso, those different preconditions were lacking and were therefore important barriers to the implementation of PBF, hence its scale-up. To disregard these preconditions and to remain at a more theoretical level could be due to the ownership of the project, which is mainly donor-driven (donors that may be less aware of contextual factors) and therefore less scalable, less sustainable over the long term.^72^ The Burkina Faso context is also particular in the sense that the country has been through much instability whether social (social insurrection), political (eg, end of more than 25 years of Compaoré’s regime) or institutional (eg, the constant change at the head of the MoH). The issue of corruption can easily come to mind in Burkina Faso, which was ranked 72th of 176 countries in regards to transparency.^78^ Corruption can directly affect PBF results and implementation, which can then negatively influence the prospect of scaling-up. For example, low salaries as well as delays in payments can create incentive for corruption from healthcare workers or the impunity of corrupt individuals at higher levels (eg, district chief doctor) can install an omertà in the organisations.^78^ Finally and most interestingly, the final impact evaluation of PBF in Burkina Faso was still not published, bringing thoughts about a potential lack of results, what administrative data analyses seem to show at the moment.^54^ All those elements can give a bad perception of the intervention, affecting the decision-making process on scaling-up.

###  Limits

 Some limits must however be addressed such as the use of the specifically tailored framework to assess PBF scale-up operations.^21^ This would have led to generating more comparable results between the burkinabè’s case and the different programs cited-above. However, data collection was already finished, based on the ExpandNet’s framework, when this more specific framework was published. Many contextual changes also happened following the data collection phase. The support and presence of the national research team was therefore necessary to complete the picture.

 To further the validity of the results and to have a more exhaustive view of PBF in Burkina Faso, it would have been interesting to use or strengthen other data collection methods, whether qualitatively (eg, documentary research, observations) or quantitatively (eg, questionnaire). Those methods would have enabled a better triangulation of the results.^50,79^ Due to resource and time constraints, the coding and the analysis of the data was also done by only one member of the research team (MS). Discussion around the manuscript (VR, PAS) helped to gather other points of view about the results found, but a more structured and systematic approach would have helped to have a more exhaustive and reliable analysis.

 In regards to the ExpandNet framework used for this study, its format, a check-list model, can be challenging in providing a complete picture of the situation. This framework does not emphasise interactions between its different dimensions (eg, the innovation, host organisation, resource team, strategies and context) and leads to a more descriptive and linear account of the assessed scaling-up process. The AHPSR’s conceptual framework used in the discussion is responding to this challenge by taking into account the complexity of the intervention.^21^ Again however, its 4 phases for scale-up still restrains a potential dynamic analysis of scale-up. Much iterative actions and back-and-forth discussions take place during scale-up, regardless of linear phases, which are no barriers to scale-up (or not) an idea, a pilot, a project or a program.

## Conclusion

 While the PBF scale-up was assumed to be the natural step forward for the pilot, an eventual nation-wide PBF program does not seem possible in the foreseeable future, with the exception of a user fee exemption program that is the priority at the moment for the government. Lessons from the burkinabè experience should be clear, namely to implement a reform that is reformative, yes, but also adapted to its context’s needs, values or institutions. A balance must then be found between the reform’s objectives (that are often elaborated abroad, being donor-driven programs) and national priorities.^15,41^ A move from “scale-up” to “scale-out”^24^ should then be more apparent, with a greater account for contextual factors. Now the question remains of whether the use of strategic purchasing in the actual discussions for the SNFS can be thought of a PBF scale-up or not.

## Acknowledgments

 We would like to thank Ahmed Barro for his help during the data collection phase as well as the larger coordination of the project. Thanks to all the transcribers on the AGIR team for their hard work transcribing. Thanks to Christina Zarowsky for her comments on the manuscript and to Douglas Rideout for the review. This work was supported by the Canadian Institutes of Health Research (CIHR), which funded the program (ROH-115213) “Community research studies and interventions for health equity in Burkina Faso.” The sponsors had no role in the study’s design; the collection, analysis and interpretation of data; the writing of the report; or the decision to submit the article for publication.

## Ethical issues

 All participants provided written consent (oral consent concerning the observations) for the interviews. This consent was free of pressure, informed by a conversation about the research, and continued, since the interviewer could ask again for consent during the interview. Confidentiality and anonymity were guaranteed during and after the study. No incentives were given to the participants to participate. This study was approved by ethic committees from the *Centre de Recherche de l’Université de Montréal* (CE 13.358) and Burkina Faso (N° 2015-12-07).

## Competing interests

 Authors declare that they have no competing interests.

## Authors’ contributions

 VR and PAS wrote the protocol with the support of other research team members. MS collected, analysed the data and wrote the manuscript. VR and PAS supervised data collection and interpretation with recommendations. VR and PAS participated in the review of the manuscript. All have read and approved the final manuscript.

## Authors’ affiliations

^1^Université de Montréal, École de santé publique (ESPUM), Montréal, QC, Canada. ^2^Research Institute for Sustainable Development, CEPED (IRD-Université de Paris), Paris, France. ^3^AGIR (Action-Gouvernance-Intégration-Renforcement; Groupe de travail en Santé et Développement), Ouagadougou, Burkina Faso.

## Endnotes

 [1] Citations were freely translated from French by the first author who did the interviews.
